# Supplementary material for: Splice-Junction-Based Mapping of Alternative Isoforms in the Human Proteome
Source: Cell Rep. Author manuscript; Available in PMC 2020 Jan 15. (PMC6961840; doi:10.1016/j.celrep.2019.11.026)
Supplement: 2 [file NIHMS1546469-supplement-2.zip › DF1/PXD006675/Aorta/Aorta_7_YWHAZ_GDEAEAGEGGERFGVSYPGIVDQSQQAYQEAFEISK.pdf]

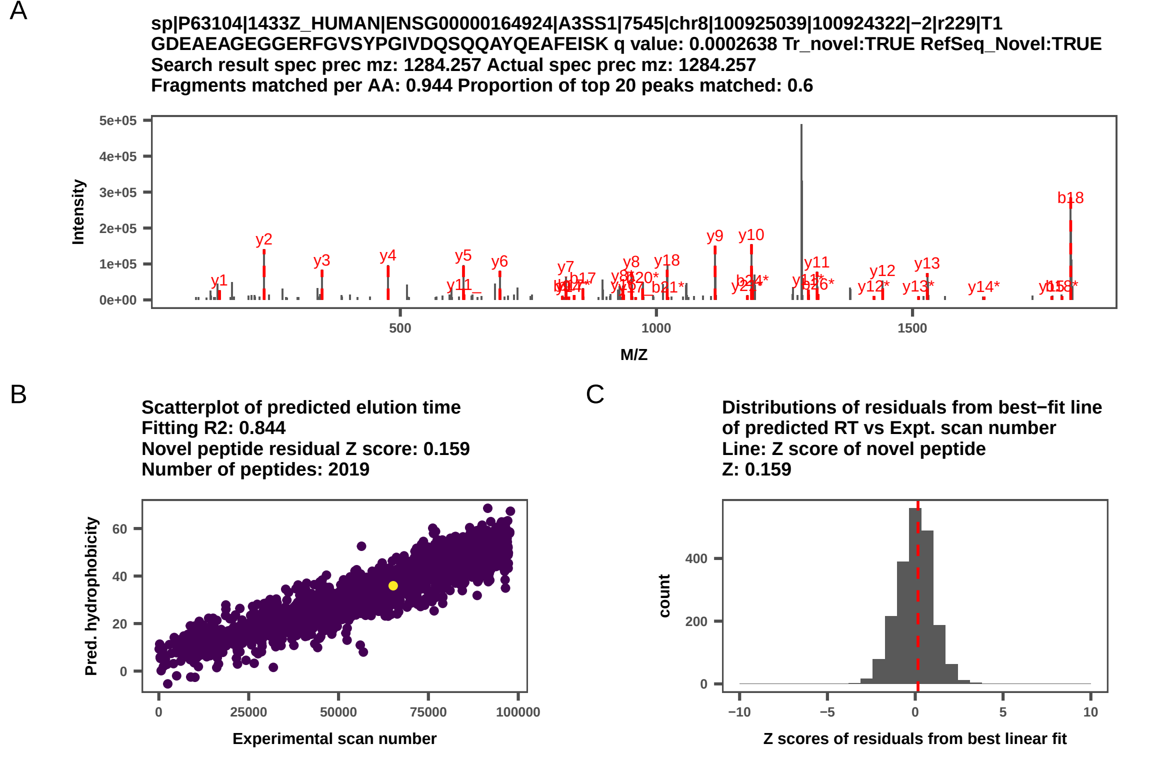
A
sp|P63104|1433Z_HUMAN|ENSG00000164924|A3SS1|7545|chr8|100925039|100924322|−2|r229|T1
GDEAEAGEGGERFGVSYPGIVDQSQQAYQEAFEISK q value: 0.0002638 Tr_novel:TRUE RefSeq_Novel:TRUE
Search result spec prec mz: 1284.257 Actual spec prec mz: 1284.257
Fragments matched per AA: 0.944 Proportion of top 20 peaks matched: 0.6
Intensity
M/Z
y1
y2
y3
y4
y5
y6
y7
y8
y9
y10
y11
y12
y13
y18
b18
y12*
y13*
y14*
b21*
0e+00
1e+05
2e+05
3e+05
4e+05
5e+05
500
1000
1500
B
Scatterplot of predicted elution time
Fitting R2: 0.844
Novel peptide residual Z score: 0.159
Number of peptides: 2019
Pred. hydrophobicity
Experimental scan number
0
20
40
60
0
25000
50000
75000
100000
C
Distributions of residuals from best−fit line
of predicted RT vs Expt. scan number
Line: Z score of novel peptide
Z: 0.159
count
Z scores of residuals from best linear fit
0
200
400
−10
−5
0
5
10
